# Detection and prognostic role of heterogeneous populations of melanoma circulating tumour cells

**DOI:** 10.1038/s41416-020-0750-9

**Published:** 2020-02-10

**Authors:** Carlos Alberto Aya-Bonilla, Michael Morici, Xin Hong, Ashleigh Cavell McEvoy, Ryan Joseph Sullivan, James Freeman, Leslie Calapre, Muhammad Adnan Khattak, Tarek Meniawy, Michael Millward, Mel Ziman, Elin Solomonovna Gray

**Affiliations:** 1 School of Medical and Health Sciences, Edith Cowan University, Perth, WA Australia; 2 Massachusetts General Hospital Cancer Center, Harvard Medical School, Boston, MA USA; 3 Department of Medical Oncology, Fiona Stanley Hospital, Murdoch, WA Australia; 4 School of Medicine, University of Western Australia, Crawley, WA Australia; 5 Department of Medical Oncology, Sir Charles Gairdner Hospital, Nedlands, WA Australia; 6 School of Biomedical Science, University of Western Australia, Crawley, WA Australia

**Keywords:** Tumour biomarkers, Melanoma

## Abstract

**Background:**

Circulating tumour cells (CTCs) can be assessed through a minimally invasive blood sample with potential utility as a predictive, prognostic and pharmacodynamic biomarker. The large heterogeneity of melanoma CTCs has hindered their detection and clinical application.

**Methods:**

Here we compared two microfluidic devices for the recovery of circulating melanoma cells. The presence of CTCs in 43 blood samples from patients with metastatic melanoma was evaluated using a combination of immunocytochemistry and transcript analyses of five genes by RT-PCR and 19 genes by droplet digital PCR (ddPCR), whereby a CTC score was calculated. Circulating tumour DNA (ctDNA) from the same patient blood sample, was assessed by ddPCR targeting tumour-specific mutations.

**Results:**

Our analysis revealed an extraordinary heterogeneity amongst melanoma CTCs, with multiple non-overlapping subpopulations. CTC detection using our multimarker approach was associated with shorter overall and progression-free survival. Finally, we found that CTC scores correlated with plasma ctDNA concentrations and had similar pharmacodynamic changes upon treatment initiation.

**Conclusions:**

Despite the high phenotypic and molecular heterogeneity of melanoma CTCs, multimarker derived CTC scores could serve as viable tools for prognostication and treatment response monitoring in patients with metastatic melanoma.

## Background

Melanoma tumour cells exhibit pronounced heterogeneity within a single tumour, amongst concurrent tumours within the same patient and amongst tumours from different patients.^1–3^ This heterogeneity is the greatest challenge in the design and management of cancer treatments, since a small proportion of clones may still remain as a result of treatment-selective pressure.^4^

Circulating tumour cells (CTCs), which are responsible for haematogenous spread of tumours, also reflect this heterogeneity. The heterogeneous expression of markers, together with their rare frequency (1–10 CTCs in 8 mL of blood) are the main intrinsic factors hampering CTC isolation and limiting in-depth study of these cells in patients with melanoma.^5–9^ Several methodologies have been developed to improve CTC isolation, the most advantageous are microfluidic platforms as these allow for target-independent enrichment of heterogeneous CTCs and with relatively low white blood cell (WBC) background (i.e. high WBC depletion rates: 3–5 log).^6,10–13^

To explore the heterogeneity of CTCs, a number of antibody-based and transcript-based assays have been developed for detection of a broader range of melanoma CTCs.^7–10,13,14^ In particular, we have shown the heterogeneous expression of five melanoma-specific genes used to identify CTCs isolated using the slanted microfluidic device or immunomagnetic enrichment.^10,15^ Recently, Hong et al. identified the molecular signatures of melanoma CTCs, after enrichment using the CTC-iCHIP.^13^ Based on the expression of 19 genes a digital quantitation method (CTC score) was developed, and this score was used to enable monitoring of early response to immune checkpoint inhibitors in individuals without traceable mutations in the tumour (i.e. *BRAF* loci).

In this study, we compared the recovery and purity of melanoma cells isolated through different microfluidic devices, Parsortix (Angle plc, Surrey, United Kingdom) and ClearCell® FX1 system (Biolidics, Singapore), which enrich CTCs based on their differential cell size, density and deformability. Using CTC fractions isolated by Parsortix (Angle plc), we investigated the molecular heterogeneity of CTCs isolated from 43 blood samples from metastatic melanoma patients. CTC were detected by multiplex immunostaining and multi-transcript detection.^10,13^ In a subset of patients, CTC detection was compared to patient/sample-matched ctDNA levels and clinical outcomes were assessed.

## Methods

### Cell culture

The melanoma cell lines A2058 and SK-MEL-5 were used in spiking experiments due to their differential cell size.^10^ Both cell lines were originally obtained from the American Type Culture Collection (ATCC, USA) and were cultured as previously described.^10^ Cell lines were harvested between 50% and 80% cell confluence using a gentle disassociation agent (TrypLE™ Express, Thermo Fisher Scientific, Waltham, MA). Only cell cultures with a cell viability higher than 90% were used in downstream applications. Cell viability was determined by Trypan Blue staining on an automated Vi-CELL XR cell counter (Beckman Coulter).

### Healthy volunteers and metastatic melanoma patients

Healthy volunteers and melanoma patients signed consent forms approved by the Human Research Ethics Committees at Edith Cowan University (No. 11543) and Sir Charles Gairdner Hospital (No. 2013–246). The study was performed in accordance with the Declaration of Helsinki. Peripheral blood samples from healthy donors and melanoma patients were collected in 9 mL BD Vacutainer K2 EDTA tubes (BD Biosciences) by phlebotomists and processed within 24 h. Melanoma patients with metastatic disease, but prior to clinical treatment with either MAPK or immune checkpoint inhibitors were included in this study (Table S1). Radiographic imaging was performed per standard of care, approximately at 3 months intervals or when clinically appropriate. Response to treatment was defined by the treating clinicians.

### Melanoma cells and CTC enrichment

A total of 8 mL from healthy donors spiked with melanoma cells and from patients with melanoma were processed on the Parsortix™ System (Angle plc) platform using a 6.5 µm cassette, following manufacturer’s guidelines. Enriched CTC fractions were harvested in 200 µl volumes.

Blood samples were also processed using the ClearCell® FX1 system (Biolidics), as indicated by the manufacturer. Briefly, 7.5 mL of blood was lysed by adding 22.5 mL of red blood cell lysis buffer, followed by a 10 min incubation and subsequent centrifugation at 500 g for 10 mins. Cells were resuspended in 4 mL of ClearCell Resuspension Buffer and loaded into the ClearCell system for CTC isolation on a primed spiral chip. Spiked samples were processed using both low (LP) and high purity (HP) protocols, whilst the low purity protocol was used to isolate CTCs from melanoma patients’ blood samples.

For samples from melanoma patients, two 8 mL blood samples were processed per patient for each platform. The first one underwent multimarker immunostaining for phenotypic CTC detection and the second one was used for RNA/DNA isolation for downstream molecular CTC detection.^10^

### Spiking of melanoma cells into blood from healthy donors

Prior to spiking, A2058 and SK-MEL-5 cell lines were respectively stained with CellTracker™ Red CMTPX and Green CFMDA dyes (Thermo Fisher Scientific), as per manufacturer’s guidelines. Blood samples from healthy donors were co-spiked with 50 pre-labelled cells from A2058 and SK-MEL-5 cell lines, using a manual picking and spiking method. Experiments were performed in triplicate for each platform.

For melanoma-spiked samples processed through the Parsortix and ClearCell platforms, capture rates were determined by counting CellTracker-labelled cells captured in the cassette in an inverted fluorescent microscope (Eclipse Ti-E, Nikon®). After processing through both platforms, CTC fractions were collected into a 96-well flat µClear® bottom plate (Greiner-Bio) and nuclei were stained by adding a drop of NucBlue™ Live ReadyProbes™ Reagent (Thermo Fisher Scientific), followed by incubation at 37 °C for 30 mins. Well plates were automatically scanned using an Eclipse Ti-E, Nikon® microscope and the NIS-Elements High Content Analysis software, version 4.2 (Nikon®). CellTracker red- and green-labelled cells were counted manually to determine recovery rates for each cell line per platform. To determine the total WBC background for each CTC fraction and platform, blue-stained nuclei were automatically counted, using a protocol developed in the NIS software. LP and HP protocols on the ClearCell were performed on paired healthy donor blood samples.

### Immunostaining

For detection of CTCs in the CTC fractions yielded after processing of blood samples from melanoma patients through the Parsortix and the ClearCell platforms, we employed a slightly modified version of the methodology described by Aya-Bonilla, Marsavela (10). Briefly, cells within the CTC fractions were fixed by gentle addition of 190 μL of ice-cold 4% Paraformaldehyde (PFA), for a final ~2% PFA concentration, following incubation for 10 mins in a BSA-coated cytofunnel, before being spun onto a Cytoslide™, fixed cells were gently transferred to a BSA-coated cytofunnel, before cytospining the cells onto a glass slide at 2000 rpm for 5 mins (Cytospin™ 4, Thermo Fisher Scientific). Cells were permeabilised by incubating for 10 mins in a 0.2% Triton X-100 (TX-100) PBS solution and blocked after incubation in a blocking solution containing 10% normal donkey serum (NDS), 3% bovine serum albumin (BSA) and 0.2% Triton X-100 (TX-100) in PBS for 10 mins. Following steps as previously detailed,^10^ cells were stained using a cocktail of the following antibodies: rabbit anti-gp100 (clone EPR4864, Abcam), rabbit anti-MLANA/MART-1 (clone EP1422Y, Abcam), rabbit anti-S100 (EP1576Y, Abcam), mouse anti-MCSP conjugated to Alexa Fluor® 647 dye (clone 9.2.27, BD Pharmingen™) and mouse anti-CD45 conjugated to Phycoerythrin (PE) (clone HI30, BD Pharmingen™). A donkey anti-rabbit IgG antibody conjugated to Alexa Fluor® 488 dye (Abcam) in blocking buffer was used to detect the intracellular melanoma antibodies. Finally, cells were stained with DAPI (4′,6-diamidino-2-phenylindole) nuclear stain and mounted using ProLong® Gold Antifade Mountant with DAPI reagent (Thermo Fisher Scientific). Slides were stored at 4 °C, visualised and scanned using the Eclipse Ti-E inverted fluorescent microscope (Nikon®). Stained cells were analysed using the NIS-Elements High Content Analysis software, version 4.2 (Nikon®) by two trained researchers. Cells with DAPI staining and positivity for any melanoma staining (i.e. MCSP or MEL staining) but negative for leucocytic-specific CD45 staining were deemed as melanoma CTCs. WBC background for each patient was determined by reliably counting all the DAPI-stained nuclei on the slide, using a protocol for automatic counting on the NIS-Elements High Content Analysis software, version 4.2 (Nikon®).

### Reverse transcription-PCR

Isolation of total RNA and gDNA, cDNA synthesis and targeted preamplification on CTC-enriched fractions were performed as previously described^16^ using TaqMan assays (Thermo Fisher Scientific) for *MLANA* (Melan-A), *TYR* (Tyrosinase), *MAGEA3* (melanoma antigen family A3), *ABCB5* (ATP-binding cassette subfamily B member 5) and *PAX3* (paired box protein Pax-3 isoform 3). The 18S ribosomal RNA gene was used as an endogenous control. Cycling conditions and fluorescence detection of these PCR reactions were performed in a ViiA™ 7 Real-Time PCR system (Thermo Fisher Scientific) as previously described.^16^ Parsortix-enriched CTC fractions from healthy controls, unspiked and spiked with melanoma cells, are respectively included as negative and positive controls in each Reverse transcription-PCR (RT-PCR) run, as described originally.^10^ Detection of transcripts across healthy controls processed through Parsortix are shown in Supplementary Table 2 (Table S2).

### Droplet digital PCR

Additional gene expression profiling of CTC fractions isolated from metastatic melanoma patients using the Parsortix platform was performed using a ddPCR expression assay, recently described by.^13^ In this assay, transcript levels from 19 genes (*MCSP* (*CSPG4)*, *FAT1*, *FAT2*, *GAGE1*, *GPR143*, *IL13RA2*, *MAGEA1*, *MAGEA2*, *MAGEA4*, *MAGEA6*, *MAGEC2*, *MLANA*, *PMEL*, *PRAME*, *SFRP1*, *SOX10*, *TFAP2*, *TNC* and *TYRP1*) were quantified in the CTC fractions from all the Parsortix-enriched samples. Total RNA samples of Parsortix-enriched CTC fractions from patients and controls, were reverse transcribed using SuperScript VILO. cDNA was subjected to gene-specific pre-amplification of the 19 target genes, for either 10 or 14 cycles, as detailed by Hong et al.^13^. Optimal pre-amplification cycles for each gene were determined by comparing the expression levels of each gene at 10 and 14 cycles using RNA from melanoma cell lines and from Parsortix-enriched samples from 12 healthy donors (Supplementary Material). The pre-amplified cDNA was then subjected to droplet digital PCR (ddPCR). Droplets were generated using an Automatic Droplet Generator (Bio-Rad) and read using the QX200™ PCR System (Bio-Rad, Hercules, CA). CTC scores were generated, using dilution corrected values in copies/ml of blood processed, by subtracting, for each gene, the healthy control set median plus two standard deviations from the results of the patient sample, adjusting any negative results to zero, and summing the resulting values across the 19 genes.^13^

### Circulating tumour DNA quantification

Cell free DNA was isolated from 5 mL of plasma using QIAamp Circulating Nucleic Acid Kits (Qiagen, Hilden, Germany) as per the manufacturer’s instructions. cfDNA was eluted in 40 µl AVE buffer (Qiagen) and stored at −80 °C until ctDNA quantification by mutation specific ddPCR. Driver mutations were identified in the patient’s tumour by either routine pathology testing for BRAF mutations or using a custom melanoma-specific targeted next generation sequencing panel as described by Calapre et al.^17^. Commercially available and/or customised probes were used to analyse ctDNA by ddPCR (Bio-Rad) as described previously.^17–19^

### Statistical analyses

A Mann–Whitney *U* test was used to compare WBC background in CTC fractions between Parsortix and ClearCell (LP mode). A log-rank (Mantel-Cox) test was used to determine the association and hazard ratios (HR) of CTC scores with overall survival (OS) and progression free survival (PFS). OS and PFS were defined as the time interval for the start of therapy to death or the date of first progression, respectively. A linear regression and a Pearson’s correlation test determined the correlation between CTC scores and ctDNA levels. Results were analysed in GraphPad Prism 8 and were considered significant at a *p* < 0.05.

## Results

### Isolation of melanoma CTCs using microfluidic devices

**Fig. 1:**
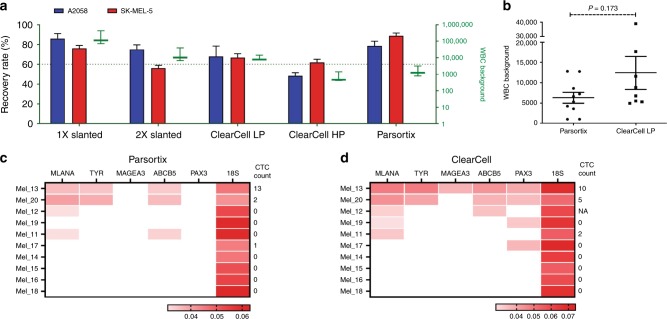
CTC isolation platforms comparison. **a** Comparison of the Parsortix (Angle plc), ClearCell FX1 (Biolidics) (LP: low purity and HP: high purity programmes) and the Slanted spiral microfluidics platforms, on their recovery rates of the melanoma cell lines A2058 or SK-MEL-5 (indicated as bars: left y-axis) and total WBC counts (indicated in green as mean ± SD: right *y*-axis). **b** WBC counts in CTC enriched samples, from 10 patients with metastatic melanoma, using the Parsortix (range: 965–12831) and ClearCell systems (LP mode, range: 4925–17732). **c** and **d** Expression levels of *MLANA, TYR, MAGEA3*, ABCB5, and *PAX3* assessed by RT-PCR in matched CTC fractions enriched by **c** Parsortix and **d** ClearCell platforms. Each heatmap squares represents the expression levels for each gene and a given sample, shown by reciprocal Ct values. The 18S rRNA gene was used as an endogenous control. The CTC counts obtained by multimarker immunostaining for each matched CTC fraction derived from the same patient are indicated. NA data not available.

We compared two commercial microfluidic platforms for the enrichment of CTCs, Parsortix and ClearCell. For comparison, we also included data from the previously described slanted spiral microfluidic device for enrichment of CTCs.^10^ Blood samples from healthy controls spiked with 50 cells each from A2058 and SK-MEL-5 cell lines were used to determine their recovery rates and total WBC background (Fig. 1a). Since ClearCell has two types of enrichment protocols, high and low purity, we included both in our comparison. From all these enrichment systems, Parsortix yielded the highest recovery (A2058: 79%; SK-MEL-5: 89%) at a low total WBC count (mean: 1696; range: 800–3064). The HP enrichment on ClearCell yielded a recovery of 49% for A2058 and 62% for SK-MEL-5, with the lowest total WBC background (mean: 767; range: 423–1414). In contrast, the ClearCell on LP mode had a similar recovery for both cell lines (A2058: 68%; SK-MEL-5: 67%) but a higher WBC background of 9939 WBCs (range: 7752–14273). These values show a significant improvement in the WBC depletion ability of these two systems, in comparison to those obtained previously^10^ using the slanted spiral microfluidic device (A2058 recovery: 75%, SK-MEL-5 recovery: 56%; WBC range: 6750–38,250) (Fig. 1).

To further compare the two platforms; Parsortix and ClearCell (LP), we analysed matched blood samples from 10 metastatic melanoma patients. Consistent with the results from healthy controls, Parsortix yielded on average two-fold lower WBC background than the ClearCell (Fig. 1b). For detection of CTCs after enrichment we utilised a multimarker melanoma-specific immunostaining as previously described.^10^ Using this approach, we detected CTCs in 30% of patients from both Parsortix (range: 1–13 CTCs) and ClearCell (range: 2–10 CTCs) (Fig. 1c, d). However, two cases had detectable CTCs in only one of the platforms (Mel_17, 1 CTC after Parsortix and Mel_11, 2 CTCs after ClearCell enrichment).

We also analysed the enriched fractions for expression of five melanoma-specific genes, assessed by RT-PCR as previously described.^10^ We detected at least one melanoma transcript in 40% and 60% of samples enriched by Parsortix and ClearCell, respectively, with two samples only positive in the ClearCell-enriched fraction (Mel_17 and Mel_19) (Fig. 1c, d). ClearCell-enriched fractions also showed a slightly higher abundance of detected transcripts compared to Parsortix-derived samples.

Overall, similar detection rates were observed using Parsortix and ClearCell LP, however, Parsortix yielded lower WBC backgrounds providing a more suitable platform for transcriptomic analysis. Thus, follow-up analyses were carried out in samples enriched using the Parsortix platform.

### Heterogeneous expression of melanoma-specific genes in CTC enriched fractions

**Fig. 2:**
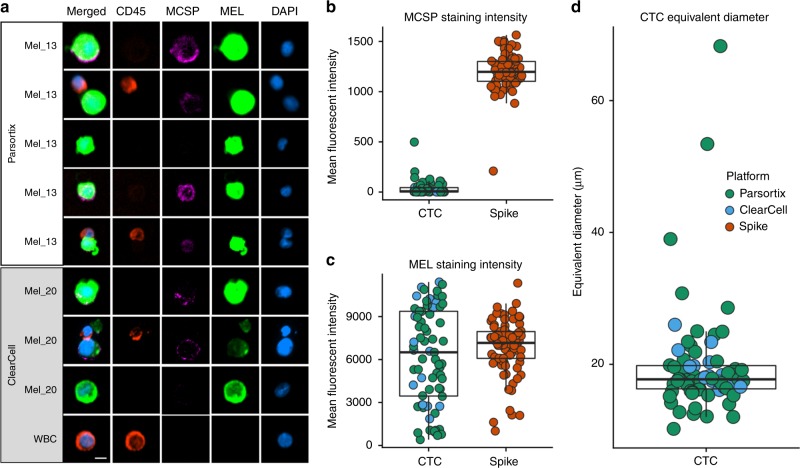
Phenotypic and morphological heterogeneity of melanoma CTCs after microfluidic enrichment. **a** Representative CTCs isolated from melanoma patients using Parsortix (Angle plc) or ClearCell FX1 (Biolidics). CTCs were identified through positive immunostaining for MCSP (Alexa Fluor® 647, purple) and intracellular -MEL (gp100, S100 and MLANA; Alexa Fluor® 488, green) markers. DAPI was used for nuclei staining and CD45 (PE, red) as a leucocytic marker. Immunofluorescence intensity of patients CTCs for **b** the cell surface marker MCSP and **c** the combination of the melanoma-specific intracellular markers (MEL staining). CTCs recovered with either device are indicated in different colours. The immunofluorescence intensity of A2058 cells spiked into healthy donors’ blood, recovered and detected using the same methods used for patient samples, were included as a reference and labelled as “spike". **d** Cell size distribution of melanoma CTCs. Merged images of the two largest CTCs observed were included. Graphs are represented as scatter plots with median and interquartiles shown as box and whiskers plots. Scale bars indicate 10 µm.

**Fig. 3:**
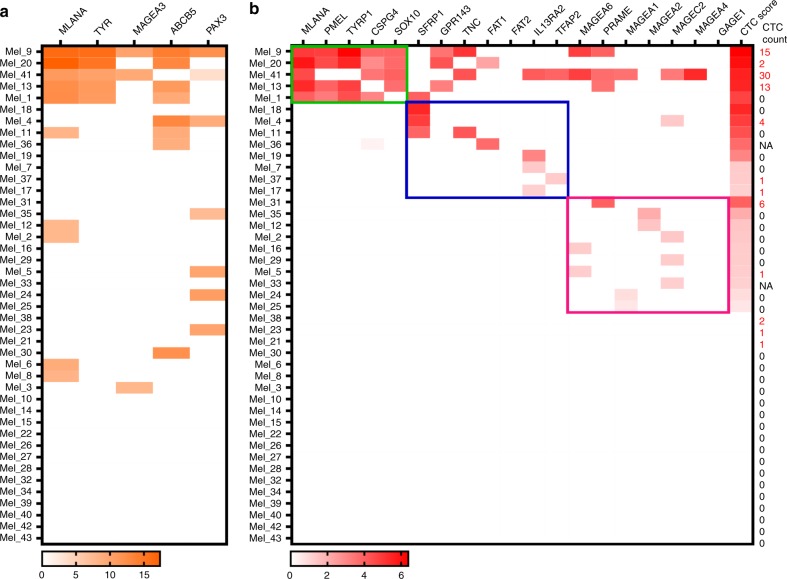
Comparison of gene expression profiles of CTC fractions using the five melanoma-specific RT-PCR and the 19 genes ddPCR. Number of CTCs detected by a multimarker immunostaining protocol of their respective matching sample is indicated to the right and positive CTC counts are shown in red on the far right of figure. The genes with positive detection by RT-PCR (**a**, orange) or ddPCR (**b,** red) are shown for each CTC fraction, and the colour of each box represents the expression level for a particular gene and CTC fraction (log scale—indicated below). CTC scores from the ddPCR **b** are also indicated. Colour boxes indicate samples positive for genes categorised either as a melanocyte lineage (green), cancer-related (blue) or cancer testis antigen (pink). NA data not available.

To characterise the molecular makeup of CTCs enriched by Parsortix, we compared CTC fractions derived from 43 blood samples from 37 melanoma patients. Patient characteristics were described in Table S1. For these samples, we obtained a median of 8386 WBCs (range: 965–119,183), while one patient had unmeasurably large WBC counts in the CTC fraction. CTCs were detected by immunocytochemistry in 12 out of 43 samples (28%) (range: 1–30 CTCs) (Figs. 2 and 3). We observed a large variability in the immunofluorescence intensity values of the multimarker intracellular staining (MEL) and the cell surface marker MCSP across the detected CTCs (Fig. 2a–c). Additionally, CTCs were found to be variable in size ranging from 10.2 to 68.3 µm, however, most of them clustered around a median size of 17.7 µm (Fig. 2d). These findings strongly indicate the morphological and phenotypic heterogeneity of melanoma CTCs.

Gene expression analysis of five melanoma-associated genes, including *MLANA, TYR, MAGEA3, ABCB5* and *PAX3*, showed that at least one transcript was detected in 18 out of 43 samples analysed (42%) (Fig. 3a). Of note, only 7 out of these 18 cases (39%) had detectable CTCs by immunostaining. Overall, *MLANA* was the most commonly detected transcript (*n* = 10), while *TYR* (*n* = 5) and *MAGEA3* (*n* = 3) were the least detected. Exclusive expression of either *ABCB5* or *PAX3* was found in 7 out of 18 transcript-positive cases (39%). Co-expression of any of these stem cell markers with other genes was observed in an additional 6 CTC fractions, with a total of 13 out of the 18 (72%) CTC positive fractions showing expression of at least one melanoma-initiating marker.

### Extended gene expression analysis of 19 genes in CTC enriched fractions

We assessed the expression of 19 transcripts using ddPCR as recently described by Hong et al.^13^ and determined a CTC score for each sample. We found that 23 out of 43 CTC fractions (53%) were positive for at least one transcript (Fig. 3b and Table S3). The mean CTC score for positive samples was 106,125 transcript counts per mL of blood (range: 4–2.4 × 10^6^), with 12 out of 43 CTC fractions (28%) having CTC scores above 100 transcript counts per mL. Patients with high CTC scores commonly had two or more genes detected.

We segregated the CTC positive samples based on the group of genes more highly represented (Fig. 3b). Samples positive for melanocytic lineage genes were strongly positive with at least three co-expressing genes. In contrast, fractions expressing cancer-related or cancer testis antigen genes only were usually positive for only one gene and had low number of transcripts detected.

### The combination of multimarker phenotypic and molecular assays improves CTC detection rates

CTC detection rates varied between methodologies with only 28% of samples positive by immunostaining, 42% using the 5-genes RT-PCR assay and 53% using a 19-genes ddPCR assay. By combining the detection rates obtained from immunostaining and molecular assays, 31 out of 43 CTC fractions (72%) were positive by at least one assay (Fig. 3). Of those, only six samples were positive by all three assays, and only four with two or more CTCs detected, high CTC scores and more than three transcripts detected by RT-PCR and ddPCR. In contrast, patient Mel_4 had four CTCs detected by immunostaining but was negative for melanocytic lineage markers in the transcript analyses, positive for *SFRP1* by ddPCR and *PAX3* and *ABCB5* by RT-PCR. CTC fractions with only one CTC detected displayed no or low CTC scores. Of the CTC fractions with no CTCs detected by immunostaining, Mel_1, Mel_11, Mel_18 and Mel_19 showed high CTC scores; particularly, we highlight Mel_1 and Mel_11 due to the co-detection of more than two transcripts by ddPCR and RT-PCR. Of note, only two CTC fractions (Mel_21 and Mel_38) had 1 or 2 CTCs detected by immunostaining but no transcripts detected by ddPCR or RT-PCR.

Contrasting of the genetic profiles of CTC fractions analysed by ddPCR and RT-PCR revealed the molecular diversity displayed by melanoma CTCs. Also, correlation in the detection of melanocytic lineage markers was observed in five CTC fractions, confirming a strong melanocytic transcriptional programming of some melanoma CTCs. Of note, *TYR* transcripts detection by RT-PCR correlated with *TYRP1* detection by ddPCR. *MLANA* is the only gene shared between the RT-PCR and ddPCR gene panels. Five CTC fractions had *MLANA* transcripts detected by RT-PCR, but not by ddPCR. It is possible that very low transcript concentrations fail to efficiently amplify when a high level of multiplexing is performed.

### Expression of melanocytic lineage signature identifies CTC subgroups

Comparison of phenotypic and molecular signatures across CTCs allowed us to segregate the CTC positive samples in three groups based on the expression of linage defining genes (Fig. 3). Samples positive for melanocytic lineage genes were strongly positive with at least 4 co-expressing genes. This melanocytic signature was featured by the high expression of *MLANA*, *PMEL*, *TYR*, *TYRP1*, *SOX10* and *CSPG4*. Other genes, such as *ABCB5*, *PAX3*, *GPR143* and *PRAME* were also commonly detected in the melanocytic samples.

In contrast, fractions expressing cancer-related or cancer testis antigen genes were usually positive for only one gene and had lower number of transcripts detected. Samples expressing cancer-related genes had higher CTC scores than cancer testis antigen positive fractions. In particular, *SFRP1* was strongly expressed and commonly accompanied by *ABCB5* expression.

### CTC scores are significantly associated with shorter OS

**Fig. 4:**
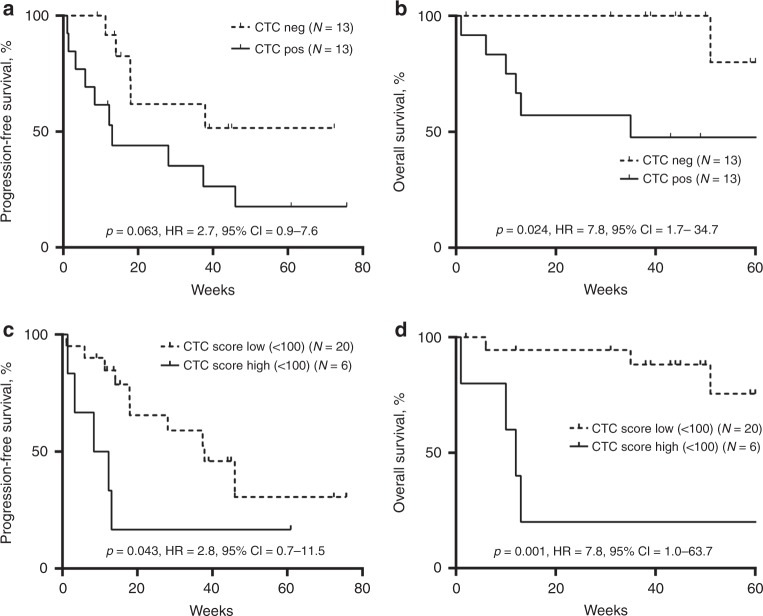
Association between CTC detection and clinical outcomes. Kaplan–Meier plots for progression-free survival (**a**, **c** and **e**) and overall survival (**b**, **d** and **f**) of patients with CTC detected using the 19-gene ddPCR assay. **a**, **b** Patients were dichotomised as positive if any of the transcripts were detected above background. **c**, **d** *P*-values, Hazard ratios and 95% confidence intervals were derived using a log-rank statistical test. Values are indicated on each graph and in bold when statistically significant.

In order to assess the prognostic value of CTCs detected here, we analysed a subgroup of patients sampled before therapy initiation (*n* = 26), with MAPK (dabrafenib/trametinib, *n* = 13) or checkpoint inhibitors (ipilimumab/nivolumab (*n* = 6) or pembrolizumab (*n* = 7)). Median follow-up time was 45 weeks (range: 12–76 weeks). CTC detection was not associated with PFS (Fig. 4a), but patients with a positive CTC score had shorter OS compared with those with undetectable CTC score (HR: 7.8, 95% CI: 1.7–34.7, *p* = 0.024) (Fig. 4b). Moreover, patients with CTC scores over 100 transcripts per mL of blood had not only significantly shorter OS (HR: 7.8, 95% CI: 1.0–63.7, *p* = 0.001), but also shorter PFS (HR: 2.8, 95% CI: 0.7–11.5, *p* = 0.043) (Fig. 4c, d).

### CTC scores are significantly associated with plasma ctDNA levels

Numerous studies have demonstrated the clinical utility of ctDNA as a biomarker for monitoring response and disease progression in melanoma patients.^19–22^ However, disease monitoring through ctDNA for patients without hot-spot mutations requires identification of a patient specific tumour-mutation, making the process time consuming and expensive.

**Fig. 5:**
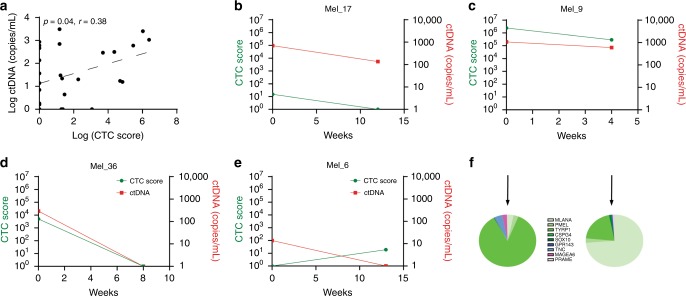
CTC scores and ctDNA comparison. **a** Correlation of CTC scores with ctDNA levels in patients with metastatic melanoma. p-value and Pearson r are indicated. **b–e** CTC scores and ctDNA changes between baseline and the first follow-up time point after initiation of treatment with Ipilimumab/Nivolumab (Ipi/Nivo) (**b**, **c**) or Dabrafenib/Trametinib (Dab/Tra) (**d**, **e**). Green and red lines and dots represent CTC score (transcripts/mL) or ctDNA levels (copies/mL), respectively. **f** Pie charts show the relative distribution of transcripts at baseline and week 4 for Mel_9. The colour of the wedges indicates the gene category (melanocytic—green, cancer-related—blue or testis antigen—pink).

We evaluated plasma ctDNA levels in 30 of the 43 patients analysed, for which a tumour-associated mutation could be identified. The median of ctDNA levels across patients with detectable ctDNA was 114 copies/mL (range: 1.6–3075 copies/mL). Plasma ctDNA levels were significantly correlated to the CTC scores (Fig. 5a). However, six cases with detectable ctDNA (>10 copies/mL) had no detectable CTCs by ddPCR (CTC score = 0). Of note, three of these six cases were positive for CTCs using the five genes RT-PCR assay. In contrast, we observed only one case (Mel_19) with a high CTC score (1145 transcripts/mL) derived from *IL13RA2* expression, with undetectable ctDNA.

Analysis of samples collected before treatment and at the first follow-up indicated that the dynamics of CTC scores mirrored ctDNA changes (Fig. 5b–d), except for Mel_6 (Fig. 5e) where an opposing trend was observed. However, both ctDNA levels and CTC scores were low in this patient, and therefore susceptible to sampling error. Mel_9 had high a CTC score with a strong melanocytic signature as well as high CTC counts by immunostaining prior commencing treatment (Figs. 3 and 5c). After 4 weeks post-treatment, limited changes in CTC scores as well as ctDNA levels were observed. The patient had further disease progression passing away after 13 weeks of treatment initiation. Both CTC fractions displayed a melanocytic signature (Fig. 5c, f), however, we observed a reduction or complete loss of *TYRP1*, *TNC* and *MAGEA6* and an increase of *MLANA* transcripts after 4 weeks on treatment, in comparison to the beginning of treatment. Thus, this ability to assess dynamics changes in molecular signatures upon treatment may provide insightful information on CTCs and tumour biology, which is an advantageous characteristic over ctDNA analysis.

## Discussion

Previously, melanoma CTCs have been demonstrated to be heterogeneous in morphology,^12^ phenotype^7–9^ and at the molecular level,^10,13–15,23^ but these results have been obtained in independent studies, often with small cohorts of patients and using different CTC isolation platforms. In the present study, by combining morphological, phenotypic and molecular approaches to detect CTCs isolated from the same platform (Parsortix) across a number of cases, we provide a comprehensive understanding of CTC heterogeneity.

Reported detection rates of melanoma CTCs have been extremely variable (40–87%).^8–10,13–15,24,25^ Such variability may be driven by the heterogeneous nature of melanoma CTCs, and the utilisation of targeted and untargeted isolation methods that may result in the enrichment of different CTC subpopulations.^7–10,12,13,15^ Here, by comparing isolation and detection methodologies using the same blood collection, we were able to accurately assess the CTC heterogeneity and interrogate their clinical utility. In this study, we observed low and very variable CTC detection rates for each approach individually: 28% for multimarker immunostaining, 42% for the 5-gene panel on RT-PCR and 53% for the 19-gene ddPCR assay. The above detection rates are lower than previously reported for these assays,^10,13,15^ which may be explained by the low disease burden among patients commencing treatment within our cohort. Nevertheless, by combining all the three assays, we increased our CTC detection rates to up to 72% in Parsortix-derived CTC fractions. Thus, these findings strongly indicate that a multimarker strategy will be better suited to detecting CTCs in melanoma than an individual approach. Nonetheless, consideration should be taken due to the intrinsic variable specificity and sensitivity across CTC detection methods.

Our findings highlight the need to assess the efficiency in the isolation of CTCs of available commercial platforms. Based on this and as a proof of concept, we obtained a slightly better performance using ClearCell compared to Parsortix, given the detection of melanoma-specific transcripts in two additional CTC samples. Here we found a detection rate of only 59% for a 19-gene ddPCR assay in comparison to the reported (87%) by Hong et al.^13^ using CTC-iChip-enriched populations. This suggests that CTC-iChip might be better suited to isolate melanoma CTCs, but a head-to-head comparison across platforms is needed to demonstrate this conclusively.

Based on the signatures obtained by ddPCR, we identified CTC subgroups characterised by a strong melanocytic, cancer-related or cancer testis antigen signature. These findings correlate with results from previous studies and confirm a preferential enrichment of a specific CTC subtypes based on the isolation platform.^10^ Previously, a lack of melanocytic lineage signature characterised by a signature of melanoma-initiating markers, *ABCB5* and *PAX3*, was observed in fractions enriched by the 2X slanted spiral device,^10^ whilst a melanocytic signature was more common in CTC fractions enriched by magnetic beads coated with anti-MCSP antibodies.^15,23^ Interestingly, the G-protein coupled receptor 143 (*GPR143*), a target gene of MITF and responsible for melanosome biogenesis,^26^ was also expressed in CTC fractions with evident melanocytic programming. Therefore, the expression of such melanocytic markers indicates a CTC subtype with a differentiated phenotype, which also correlates with the pigmented transcriptional melanoma state described recently.^27^ Other genes, such as the glycoprotein TNC (a promoter of cell invasiveness in melanoma)^27,28^ and the melanoma-associated cancer testis antigens, *MAGEA6* and *PRAME* were found but not distinctly expressed by this subgroup. Further investigation is needed to determine whether these distinct molecular signatures can provide information about the patient’s cancer and predict clinical outcomes.

In recent studies, expression of the melanoma stem cell markers *ABCB5* and *PAX3* have been detected in CTC fractions with either a marked absence^10^ or presence^15^ of melanocytic signature, indicating a possible CTC subtype with melanoma-initiating capabilities. Herein, *ABCB5* and *PAX3* transcripts were detected irrespectively of CTC subgroups, but slightly more common in the CTC fractions with strong melanocytic signature. In CTC fractions with cancer-related and cancer testis antigen signatures, these melanoma-initiating markers were found in a small number of CTC fractions. These findings are quite intriguing as transcriptional activation of either *ABCB5*, *PAX3* or both might not be essential for CTC release from the tumour or CTC survival, but may feature a type(s) of CTC with tumour-initiating capabilities, which aligns with previous observations.^9,10,15^ PAX3 is a master regulator of melanocytic differentiation and maintenance of stem cell phenotype and migration of melanoblasts^29,30^ and regulates melanocytic function,^31^ through transcriptional regulation of *MITF*^32^ and other target genes. On the other hand, the drug efflux transporter ABCB5 defines a rare slow-cycling population of melanoma cells with tumour-initiating, self-renewing and treatment-resistant capabilities.^33–35^ Based on this, it has been hypothesised that ABCB5 might confer the ability of CTCs to seed new tumours, as ABCB5 is the commonest marker detected in unenriched and enriched CTC fractions from melanoma patients.^9,10^ Comparison of transcriptomic profiles of MCSP- and ABCB5-enriched CTC fractions, indicated that ABCB5-captured CTCs have very distinct transcriptional programming, compared to the MCSP-captured CTCs, which were characterised by high expression of melanocytic lineage markers by RT-PCR.^15^ Altogether, these findings indicate that *ABCB5* and *PAX3* are expressed by CTCs regardless of melanocytic signature, but whether their expression have a functional role in these subtypes warrants further investigations.

Hong et al.^13^ assessed these 19 genes in iChip-derived CTC fractions and did not find association of the level of CTC score (low vs high) at baseline with either OS or PFS. However, a decline in CTC scores (ΔCTC score) at 7 weeks of post-treatment initiation was significantly associated with both OS and PFS. In the present study, CTCs were isolated using Parsortix and detected using the same 19-gene molecular signature. High CTC scores were strongly predictive of shorter OS and associated with shorter PFS. However, our study included a smaller cohort of patients than the original study (26 vs 49 patients) and included a mix of patients treated with MAPK or immune checkpoint inhibitors.^13^ Hence, the prognostic value of CTC scores should be further confirmed in larger cohorts. Similarly, previous studies have shown prognostic utility of melanoma CTCs at baseline,^24^ while others could not find association.^9,36^ More recent studies demonstrated that the specific CTCs subtypes (i.e. RANK + or PDL1 + CTCs), instead of the total CTC counts, are the ones that harbour biomarker utility.^9,37^ Altogether, these results may indicate that the prognostic value of CTCs is highly affected by the platform used for CTC isolation and detection methodology.

ctDNA has been extensively shown to be a reliable liquid biopsy biomarker in the monitoring of treatment response in metastatic melanoma patients.^17,19–22^ Nonetheless, a prior knowledge of the hotspot mutation(s) is mandatory for this ctDNA-based monitoring in order to be clinically useful for early detection of response and in the anticipation of early events of resistance. Such monitoring is unsuccessful in 20–30% of melanoma cases that lack BRAF or NRAS mutations.^1^ Here we found significant correlation between CTC scores and ctDNA levels at baseline in treatment-naïve patients and observed comparable trends between CTC scores and ctDNA in patients with follow-up samples. These findings are quite important as it is the first time that any correlation has been shown between CTCs and ctDNA, a recognised surrogate marker of tumour burden.^21,38^ Furthermore, we found that the use of CTC scores for monitoring can also provide insightful information about the biology of both tumour and CTCs, based on changes in the molecular signatures at baseline and post treatment, a complementary feature to ctDNA monitoring.

In this study, using an array of multimarker phenotypic and molecular approaches, we were able to significantly expand our understanding of the phenotypic and molecular heterogeneity of CTCs. However, despite detecting distinctive molecular signatures within CTCs, the real heterogeneity of CTCs remains to be elucidated and efforts should be made to ultimately identify CTC subpopulations with tumour-initiating and treatment-resistant capabilities. Recent advances in high throughput microfluidic technologies allow the interrogation of the transcriptome of thousands of individual cells in parallel, unlocking their differential transcriptome and unveiling cellular diversity of tissues. Such approaches are currently being used to dissect tumour ecosystems, including melanoma.^2,27,39,40^ Similarly, these platforms can be used for unbiased transcriptome profiling of CTCs after microfluidic enrichment to ultimately reveal CTC diversity.

In conclusion, we ascertain the highly heterogeneous nature of CTCs at both the phenotypic and molecular level in matched CTC fractions. By doing this, we identified molecular signatures within CTC fractions and detected CTC subgroups with strong melanocytic, cancer-related or cancer testis antigen signatures. Additionally, we found significant correlation between CTC scores and survival rates and between CTC scores and ctDNA levels. Thus, our findings support the promising clinical utility of CTC scores for metastatic melanoma prognostication and for real-time monitoring of treatment response, irrespective of their mutational status, and for asserting dynamic changes of CTC-derived molecular signatures upon treatment.

## Supplementary information

Supplemental Information

## Data Availability

Raw data is available upon request.
