# Supplementary material for: Detection and prognostic role of heterogeneous populations of melanoma circulating tumour cells
Source: Br J Cancer. 2020 Feb 10;122(7):1059–67. doi: 10.1038/s41416-020-0750-9 (PMC7109152; doi:10.1038/s41416-020-0750-9)
Supplement: Supplementary file 1 — Supplemental Information [file 41416_2020_750_MOESM1_ESM.docx]

**Supplementary Information**

**Pre-amplification conditions for droplet digital PCR for CTC score calculation**

We adapted the protocol from Hong *et al.* (10) for CTC scores calculation. Given the differences in the microfluidic devices used for CTC isolation between the CTC-iChip used by Hong *et al.* (13) and the Parsortix platform, we evaluated a cohort of healthy controls through the Parsortix platform to determine the background signal. Blood samples from 12 healthy volunteers were processed through the Parsortix and the enriched fractions, henceforth known as “CTC fractions”, were subjected to RNA isolation, cDNA synthesis and gene-specific pre-amplification. The number of pre-amplification cycles for each gene was determined by comparison of the transcript counts per mL of processed blood between 10 and 14 cycles to reduce background in healthy controls and oversaturation in patients’ samples. Consequently, genes with lower transcript counts per mL at pre-amplification cycle 10, or with no transcript counts at cycle 14, in healthy controls were subsequently preamplified, at same number of cycles, in patients’ samples (Table S2). In healthy donors, all the genes pre-amplified with 14 cycles had little or no background noise, whilst *FAT2*, *PMEL* and *SFRP1*, were commonly detected in more than half of healthy controls.

| **Supplementary Table 2.** Demographic and clinical information of metastatic melanoma patients | | | |
| --- | --- | --- | --- |
| **Characteristic** |  | **N** | **% of total** |
| **Total patients enrolled** |  | **37** |  |
|  |  |  |  |
| **Age at enrolment (years)** |  |  |  |
| Median | 66 |  |  |
| Range | 30 - 82 |  |  |
|  |  |  |  |
| **Gender** |  |  |  |
| Male |  | 27 | 73% |
| Female |  | 10 | 27% |
|  |  |  |  |
| **Melanoma type** |  |  |  |
| Cutaneous |  | 33 |  |
| Uveal |  | 3 |  |
| Acral |  | 1 |  |
|  |  |  |  |
| **Stage of disease at baseline** |  |  |  |
| **Stage IV** |  |  |  |
| M1a |  | 1 | 3% |
| M1b |  | 6 | 16% |
| M1c |  | 22 | 59% |
| M1d |  | 8 | 22% |
|  |  |  |  |
| **Mutational status of tumour** |  |  |  |
| ***BRAF* Mut** |  | **18** | **49%** |
| V600E |  | 12 | 32% |
| V600K |  | 5 | 14% |
| K601E |  | 1 | 3% |
|  |  |  |  |
| ***NRAS* Mut** |  | **3** | **8%** |
| Q61K |  | 2 | 5% |
| Q61R |  | 1 | 3% |
|  |  |  |  |
| ***BRAF/NRAS* WT** |  | **16** | **43%** |
|  |  |  |  |
| **Baseline for:** |  |  |  |
| Dabrafenib/Trametinib |  | 14 | 38% |
| Ipilimumab/Nivolumab |  | 9 | 24% |
| Pembrolizumab |  | 7 | 19% |
| Other treatments |  | 1 | 3% |
| No baseline |  | 6 | 16% |
